# Metabolomics and Self-Reported Depression, Anxiety, and Phobic Symptoms in the VA Normative Aging Study

**DOI:** 10.3390/metabo13070851

**Published:** 2023-07-15

**Authors:** Nicole Prince, Meryl Stav, Margaret Cote, Su H. Chu, Chirag M. Vyas, Olivia I. Okereke, Natalia Palacios, Augusto A Litonjua, Pantel Vokonas, David Sparrow, Avron Spiro, Jessica A. Lasky-Su, Rachel S. Kelly

**Affiliations:** 1 Channing Division of Network Medicine, Brigham and Women’s Hospital, Boston, MA 02115, USA; reapr@channing.harvard.edu (N.P.); resta@channing.harvard.edu (M.S.); remco@channing.harvard.edu (M.C.); su.chu@channing.harvard.edu (S.H.C.); olivia.okereke@mgh.harvard.edu (O.I.O.); jessica.su@channing.harvard.edu (J.A.L.-S.); 2 Harvard Medical School, Boston, MA 02115, USA; cvyas@mgb.org; 3 Department of Psychiatry, Massachusetts General Hospital, Boston, MA 02114, USA; 4 Department of Nutrition, Harvard T. H. Chan School of Public Health, Boston, MA 02115, USA; natalia_palacios@uml.edu; 5 Department of Public Health, Zuckerberg College of Health Sciences, University of Massachusetts Lowell, Lowell, MA 01854, USA; 6 Geriatric Research Education Clinical Center, Edith Nourse Rogers Memorial Veterans Hospital, Bedford, MA 01730, USA; 7 Division of Pediatric Pulmonary Medicine, Golisano Children’s Hospital at Strong, University of Rochester Medical Center, Rochester, NY 14642, USA; augusto_litonjua@urmc.rochester.edu; 8 Department of Veterans Affairs, Boston, MA 02114, USA; pantel.vokonas@va.gov (P.V.); david.sparrow@va.gov (D.S.); 9 VA Normative Aging Study, VA Boston Healthcare System, Boston, MA 02130, USA; aspiro3@bu.edu; 10 Department of Medicine, Boston University Chobanian and Avidisian School of Medicine, Boston, MA 02118, USA; 11 Department of Epidemiology, Boston University School of Public Health, Boston, MA 02118, USA; 12 Department of Psychiatry, Boston University Chobanian and Avidisian School of Medicine, Boston, MA 02118, USA

**Keywords:** metabolomics, normative aging study (NAS), brief symptom inventory (BSI), depression, anxiety, phobic anxiety, mental health, veterans

## Abstract

Traditional approaches to understanding metabolomics in mental illness have focused on investigating a single disorder or comparisons between diagnoses, but a growing body of evidence suggests substantial mechanistic overlap in mental disorders that could be reflected by the metabolome. In this study, we investigated associations between global plasma metabolites and abnormal scores on the depression, anxiety, and phobic anxiety subscales of the Brief Symptom Inventory (BSI) among 405 older males who participated in the Normative Aging Study (NAS). Our analysis revealed overlapping and distinct metabolites associated with each mental health dimension subscale and four metabolites belonging to xenobiotic, carbohydrate, and amino acid classes that were consistently associated across all three symptom dimension subscales. Furthermore, three of these four metabolites demonstrated a higher degree of alteration in men who reported poor scores in all three dimensions compared to men with poor scores in only one, suggesting the potential for shared underlying biology but a differing degree of perturbation when depression and anxiety symptoms co-occur. Our findings implicate pathways of interest relevant to the overlap of mental health conditions in aging veterans and could represent clinically translatable targets underlying poor mental health in this high-risk population.

## 1. Introduction

Mental disorders affect approximately one in five adults in the United States [1] and are more prevalent in vulnerable populations such as military veterans, who are at a higher risk of suffering from depression and anxiety disorders compared to the general population [2]. Delineating the biological factors that contribute to mental disorders can be challenging due to complicated molecular pathology and phenotypic heterogeneity [3] and our current understanding is further limited by the wide variety of prevalence estimates reported for these disorders in veteran and military populations [4,5,6,7,8]. However, there is an urgent need to understand the mechanisms that contribute to poor mental health, particularly in high-risk groups. Recent initiatives led by the National Institute of Mental Health (NIMH) have encouraged a shift away from the traditional framework, in which conditions are treated as diagnostically distinct, towards dimensional characterizations [9]. This approach is believed to provide a more comprehensive understanding of mental disorders and a more useful framework to investigate both distinct and overlapping symptoms. The Brief Symptom Inventory (BSI) is a validated, widely used instrument that measures symptoms of global psychological distress; the BSI includes nine subscales in which the levels of symptoms are characterized dimensionally [10] rather than strictly across Diagnostic and Statistical Manual of Mental Disorders (DSM) diagnostic criteria, according to the latest version outlined in DSM-5-TR. Estimating self-reported assessments of depression, anxiety, and phobic anxiety may capture a broader range of symptoms associated with these conditions.

Metabolomics reflects physiological processes as well as environmental influences [11,12], and the existing literature has identified pathway alterations and metabolomic changes related to mental health conditions such as schizophrenia [13], bipolar disorder [14], anxiety [15], and depression [16,17,18,19]. These studies have emphasized the power of metabolomics in accelerating mental health-related research and have facilitated the understanding of underlying mechanisms of disease across different psychiatric disorders [20]. However, previous work has primarily focused on single diseases and has generally not been studied in the context of symptom dimensions, which may limit the ability to interpret them with respect to the heterogeneity that characterizes the overlap of these conditions. Metabolomics is well suited to capture molecular functions that are perturbed both in distinct and overlapping dimensions of mental health and is likely to be successful in efforts towards personalized medicine approaches in this alternative framework [3]. A growing body of research suggests that measuring the associations between metabolites and mental health categorizations may enhance clinical precision in the diagnosis and treatment of mental disorders.

The VA Normative Aging Study (NAS) is a longitudinal study of men, most of whom are U.S. military veterans, designed to investigate the effects of aging on a variety of health conditions [21], including self-reported psychological symptoms measured by the BSI. Of the nine BSI subscales, three subscales—depression, anxiety, and phobia/phobic anxiety (i.e., anxiety characterized by an excessive and irrational fear of an object, activity, or situation)—capture the most clinically relevant symptoms of depression and anxiety disorders. Previous work has identified these three subscales as highly correlated and particularly relevant to this vulnerable population [22]. The aim of this study was to investigate cross-sectional relationships between metabolites and these three BSI subscales. Our hypothesis is that understanding associations between metabolites and the three BSI subscales that address depression, anxiety, and phobia/phobic anxiety will uncover novel insights into the biological mechanisms that contribute to overlaps and distinctions in these mental health conditions. Improved understanding of the underlying metabolic alterations associated with these subscales holds great potential for uncovering molecular mechanisms that contribute to depression and anxiety.

## 2. Materials and Methods

### 2.1. VA Normative Aging Study (NAS)

The VA Normative Aging Study (NAS) is a longitudinal study of aging in men based in Boston, Massachusetts. From 1961 to 1970, the NAS recruited 2280 men aged 21–81 who were free of known chronic disease [23]. NAS investigators collected self-reported medical and lifestyle history and performed routine physical examinations during follow-up visits. They also collected 7 mL samples of venous blood from each participant after fasting in a trace metal- and Pb-free EDTA tube periodically during follow up. The current study includes 405 men from the NAS metabolomics cohort [24], who additionally completed the Brief Symptom Inventory (BSI) questionnaire at the time of blood draw for metabolomic profiling. Written consent was obtained at each visit per the Veterans Affairs Boston Healthcare System (VABHS) IRB. This study was approved by the review boards of all institutions involved in data collection and analysis [24].

### 2.2. Brief Symptom Inventory

The BSI is a 53-item validated self-reported questionnaire that assesses nine primary symptom subscales and three composite indices of psychological distress experienced by the respondent in the last 30 days [25]. Items are measured on a 5-point scale of distress from 0 (not at all) to 4 (extremely), and the score for each subscale is calculated by averaging the responses to the questions answered for that subscale. The depression and anxiety subscales comprised six questions in total, and the phobic anxiety subscale comprised five questions. The scores were categorized as “abnormal” on a given subscale if the score was one standard deviation (SD) above the sample mean, as previously defined [24,26,27].

### 2.3. Metabolomic Profiling

As described previously [24], 405 men were selected for the NAS metabolomics subcohort on the basis of an available plasma sample collected between 2000 and 2008 that was suitable for metabolomic profiling (samples collected prior to 2000 were not suitable for profiling due to their storage conditions). Blood samples collected at the same visit as BSI administration were used for metabolite measurement. Metabolomic profiling of the plasma samples was conducted by Metabolon Inc. (Durham, NC, USA) as described previously [28] using high-resolution ultra-high-performance liquid chromatography coupled tandem mass spectroscopy (UPLC-MS/MS) across 4 platforms: (1) UPLC-MS/MS positive ionization mode and a C18 column for early eluting hydrophilic metabolites; (2) UPLC-MS/MS positive ionization mode and a C18 column for late eluting hydrophobic metabolites; (3) UPLC-MS/MS negative ionization mode and a C18 column optimized for elution of basic extracts; and (4) UPLC-MS/MS negative ion mode with a HILIC column for the separation of polar compounds. Raw data were extracted, peak-identified, and QC processed using Metabolon hardware and software. The compounds were identified through comparison of a Metabolon library of purified standards through retention time/index (RI), mass-to-charge ratio (*m*/*z*), and chromatographic data. The metabolites were quantified using the area under the curve and processed according to our standard processing pipeline: missing values imputed with half the minimum intensity for a given metabolite, followed by log-transformation, and Pareto scaling of the data. Metabolites of unknown identity or an interquartile range of zero after QC were considered uninformative and excluded from the analysis [24].

### 2.4. Statistical Analysis

We investigated the relationships between the three subscales of interest (depression, anxiety, and phobic anxiety/phobia) using Spearman rank correlations. We then conducted logistic regression modeling to estimate associations between clinically relevant (i.e., above the SD threshold) scores on the depression, anxiety, and phobic anxiety subscales with each metabolite, adjusting for age and BMI. From these results, we identified metabolites commonly associated with all three subscales at a nominal threshold of *p* < 0.05 and then used ANOVA to compare the normalized metabolite levels across three groups: men with non-abnormal scores; men with abnormal scores in a single given subscale; and men with abnormal scores on all three subscales of interest. Finally, we ran a maximally adjusted logistic model including age in years, BMI in m^2^/kg, self-reported physical health status, annual income (<$USD 25,000; USD 25,000–50,000; USD 50,000–75,000; or >USD 75,000), marital status (ever married or never married), smoking status (never smoker, ex-smoker, or current smoker), alcohol drinking status (non-drinker, light drinker, moderate drinker, heavy drinker, or other), and employment status (employed or unemployed/retired) in the smaller subset of men for whom all these variables were available. For all logistic models, the *p*-values were adjusted for the false discovery rate (FDR) using the Benjamini–Hochberg procedure to correct for the number of metabolite predictors, and the FDR-corrected *p*-values are available in Supplementary Tables S1 and S2; the main manuscript reports uncorrected *p*-values unless otherwise specified. All analyses were performed using R version 4.1.0.

## 3. Results

### 3.1. Sample Characteristics

A total of 405 men were included in our analysis (Table 1), including all men with suitable blood samples for metabolomic profiling. They had a mean age of 74.98 years (standard deviation [SD] 6.61 years) and a mean BMI of 27.65 kg/m^2^ (SD: 4.14 kg/m^2^). The majority were White (98.8%), ever married (93.6%), ex-smokers (77.8%), with self-reported good or excellent health (75.0%), and unemployed/retired (87.7%) at the time of blood collection used for metabolomic profiling. Overall, the majority of the participants scored below the abnormality threshold according to the BSI categorization rubric across the three subscales of interest [27]; the percentages of men with abnormal (i.e., above-threshold) scores on the three subscales were as follows: depression (10.1%), anxiety (7.9%), and phobic anxiety/phobia (5.2%).

### 3.2. Relationships across BSI Depression, Anxiety, and Phobic Anxiety Subscales

**Figure 1:**
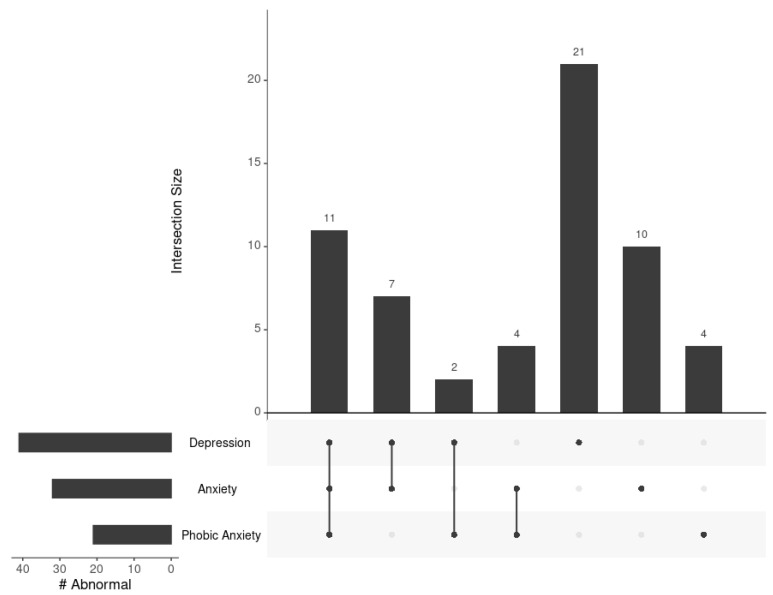
Overlap of NAS men with abnormal scores (i.e., one standard deviation above the sample mean) on the depression, anxiety, and phobic anxiety BSI subscales. In the lower left panel, the total number of men identified as having abnormal scores on each subscale is shown.

The BSI scores across these three subscales were positively correlated among the 405 men included in this sample. The strongest positive correlation was observed between depression and anxiety (*rho* = 0.58, *p* < 2.2 × 10^−16^). The correlation between anxiety and phobic anxiety showed a coefficient of *rho* = 0.49 (*p* < 2.2 × 10^−16^), and the correlation between depression and phobic anxiety was *rho* = 0.42 (*p* < 2.2 × 10^−16^). A total of 59 (14.6%) men had an abnormal score on at least one subscale. A total of 41 men had abnormal scores for depression, 32 men had abnormal scores for anxiety, and 21 men had abnormal scores for phobic anxiety (Figure 1). When examining the intersection of groups of participants with abnormal scores, 11 (2.7%) men had abnormal (i.e., one standard deviation above the sample mean) scores across all three subscales, and 13 (3.2%) had abnormal scores on two of the three subscales of interest. The remaining 35 men had abnormal scores on only one subscale: 21 (5.2%) men for depression, 10 (2.5%) men for anxiety, and 4 (1.0%) men for phobic anxiety.

### 3.3. Associations between Metabolites and BSI Dimension Abnormality

**Figure 2:**
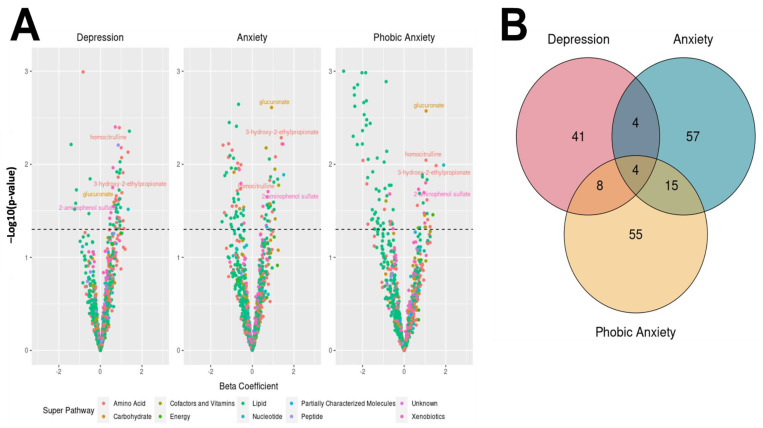
Summary of significant metabolite associations and abnormal scores on each BSI subscale colored according to the chemical class of the metabolite. (**A**) The volcano plots summarize the associations between the metabolites and each BSI subscale; the metabolites above the dotted black line met a nominal *p* < 0.05 threshold for associations with score. Metabolites belonging to sub-pathways common to abnormal scores across multiple subscales are labeled. (**B**) A Venn diagram depicts the overlap in the nominally significant metabolites at *p* < 0.05 for each of the three BSI subscales.

A total of 850 metabolites were included in the logistic models following the QC procedures. After adjustment for age and BMI, a total of 57 (6.7%), 80 (9.4%), and 82 (9.6%) of these 850 metabolites were associated with abnormal scores on the depression, anxiety, and phobic anxiety subscales, respectively, at a nominally significant *p*-value of <0.05 (Figure 2A, Supplementary Table S1). None of these associations remained statistically significant after FDR correction. Of the nominally significant metabolites for each subscale, 41 (4.8%) were uniquely associated with the abnormal depression scores (i.e., not significantly associated with anxiety or phobic anxiety scores); these metabolites spanned 29 biological pathways. A total of 57 (6.7%) were uniquely associated with the anxiety scores (i.e., not significantly associated with depression or phobic anxiety scores); these metabolites belonged to 41 biological pathways. Finally, 55 (6.5%) metabolites were associated uniquely with the phobic anxiety scores (i.e., not significantly associated with the depression or anxiety scores), including 35 biological pathways.

A total of 4 (2.1%) of the 184 metabolites associated with at least one subscale were nominally significantly associated with all three subscales (Figure 2B, Table 2). Two of these were amino acids, one was a xenobiotic metabolite, and one was a carbohydrate metabolite. The presence of abnormal scores on both anxiety and phobic anxiety/phobia subscales shared the most common hits (*n* = 19). There were 8 common metabolites between the anxiety and depression subscale scores and 12 common metabolites between the depression and phobic anxiety subscale scores. Correlations between the levels of the four metabolites in the NAS cohort demonstrated weak associations, ranging from 0.06–0.49 (Supplementary Figure S1). Aside from these commonly associated individual metabolites, there were 13 metabolite sub-pathways identified to have at least 1 metabolite significantly associated with abnormal scores on each subscale (Table 3). Within these 13 pathways, 35 metabolites were significantly associated with abnormal depression scores, 32 metabolites were significantly associated with abnormal anxiety scores, and 45 metabolites were significantly associated with abnormal phobic anxiety scores.

### 3.4. Metabolite Levels between the Abnormality Groups

Of the four metabolites commonly associated with abnormal scores on the depression, anxiety, and phobic anxiety subscales, three demonstrated a nominally significant difference (*p* < 0.05) among the five groups (Table 4): glucoronate, homocitrulline, and 2-aminophenol sulfate. All three of these metabolites showed higher relative levels in men abnormal in all three domains compared to any other group.

### 3.5. Sensitivity Analyses

We also ran a fully adjusted model including age, BMI, health status, income, marital status, smoking status, drinking status, and employment status to investigate the robustness of our primary findings in 339 men with complete data for all of the previously mentioned covariates. A total of 57, 62, and 71 metabolites were associated with abnormal scores on the depression, anxiety, and phobic anxiety subscales, respectively (Supplementary Table S2). There were 2 common metabolites between the depression and anxiety subscales, 7 between the depression and phobic anxiety subscales, and 12 between the anxiety and phobic anxiety subscales (Supplementary Table S2). Only one metabolite was associated with all three subscales in the maximally adjusted models: glucuronate. Comparisons of the betas and *p*-values in the minimally and fully adjusted models between the three subscales (Supplementary Figures S2–S4) suggest that several metabolites are no longer statistically significant following additional adjustment for potential confounders.

## 4. Discussion

The application of metabolomics to the study of mental disorders has long focused on a single clinical diagnosis, comparing cases to controls [13,14,15,16,17,19], or discriminating between two diagnoses [29]. While these studies have greatly contributed to our understanding of the biological mechanisms that underlie mental health conditions, they primarily describe metabolomic pathways and their relevance to a single condition. In this investigation, we sought to understand the overlaps in three related mental health subscales of the BSI—depression, anxiety, and phobic anxiety—to highlight metabolomic perturbations that commonly arise. Thus, our work applies a new framework to study the metabolomics of mental health by focusing on the spectrum and overlap of shared symptoms [3]. We found four metabolites across amino acid, carbohydrate, and xenobiotic classes that were consistently associated with abnormal scores in the depression, anxiety, and phobic anxiety BSI subscales. Improved understanding of the biological pathways and metabolites that contribute to depression and anxiety may lead to better diagnosis and management of these conditions, which represent the most prevalent mental health conditions [30], particularly in high-risk populations such as aging military veterans.

In investigating the potential overlap, we found two amino acid metabolites, 3-hydroxy-2-ethylpropionate and homocitrulline, consistently associated with the BSI depression, anxiety, and phobic anxiety subscales at a nominally significant level. These metabolites covered two pathways, and perturbations to these pathways have previously been observed in mental health outcomes [31]. Metabolomic studies have implicated alterations to leucine, tyrosine, and methionine pathways in mental health conditions such as anxiety and depression [32]. Likewise, our study found evidence that may suggest that higher levels of the leucine metabolite 3-hydroxy-2-ethylpropionate were associated with abnormal scores across all three BSI subscales of interest. There were four additional leucine metabolites following this pattern. While there was a lack of overlap in individual metabolite associations, our results could imply a role for methionine and tyrosine metabolism pathways, suggesting that higher levels of metabolites belonging to these amino acid pathways are also associated with worse symptoms of depression and anxiety. Changes in circulating levels of these metabolites could be representative of the systemic impacts of mental health on multiple organ systems, as observed previously in the literature. Urea cycle metabolism has also been linked to a higher prevalence of mental health conditions, and one metabolite of this pathway, homocitrulline, showed a positive association with abnormal scores on all three subscales; the accumulation of this metabolite may have negative impacts on mental health. Urea cycle disorders have been shown to have neurological implications [33] and may negatively affect mental health. Furthermore, homocitrulline levels were significantly higher in men who had abnormal scores on all three subscales, compared to those who had abnormal scores on any one subscale or who were in the control group. This could suggest that men suffering from co-morbid depression and anxiety may show increased perturbation of this metabolite. Ornithine, urea, N-methylproline, and dimethylarginine also demonstrated significant associations with at least one subscale and were generally consistent with the pattern of accumulation of urea cycle metabolites associated with worse mental health.

One xenobiotic metabolite, 2-aminophenol sulfate, and one carbohydrate metabolite, glucuronate, were nominally significantly associated with the overlap of the depression, anxiety, and phobic anxiety BSI subscales, and the directions of association indicated that accumulation of these metabolites could be related to abnormal scores. Aminophenols are often metabolites of various toxic substances, suggesting higher exposure to fungicides and pesticides [34] that are harmful to human health. Veterans in particular have been studied in the context of increased pesticide exposure [35] and its many long-term health effects. Thus, our findings could provide additional evidence in support of the possibility that increased levels of these metabolites, such as 2-aminophenol sulfate, are associated with poorer health across several dimensions of mental health symptoms. Glucuronate as a metabolite alone is difficult to assign to a single biological pathway, due to the variety of glucuronidation mechanisms employed to detoxify substances [36]. However, the findings in this study could be reflective of increased phase II metabolism mechanisms in response to increased exposure based on associations that met nominal significance [37]. This could be reflective of increased exposure to toxic substances, such as the aminophenol metabolite identified, that are correlated with poor mental health. However, it was not possible to derive which pathway could be contributing to the relationship observed between glucuronate and the abnormal dimension scores. A major limitation of our dataset was the absence of information regarding medication used that could influence the detoxification mechanisms.

Our findings also observed an overlap in four lipid pathways; while individual metabolite associations were not consistent for these pathways, lipid metabolism has repeatedly been implicated in mental health metabolomic studies [38,39,40]. This investigation found that metabolites associated with acyl carnitines, dicarboxylated fatty acids, phosphatidylethanolamines, and sphingomyelins showed associations with abnormal scores in each of the three BSI subscales of interest. Higher levels of dicarboxylated fatty acid and phosphatidylethanolamine metabolites were consistently associated with abnormal scores across the BSI depression, anxiety and phobia subscales, though individual metabolites associated with each subscale varied. Both dicarboxylated fatty acids [41] and ethanolamines [42] represent diet-derived metabolites, and alterations to these pathways may be reflective of a poorer diet associated with worse mental health scores. Lower levels of sphingomyelins were also consistently observed across groups of men who had abnormal scores for the BSI subscales but were particularly notable for phobic anxiety, which showed the most associations with this pathway. Sphingolipid metabolism has been implicated in several mental disorders [43,44,45,46], and the inverse associations observed in this study may suggest a disruption in the balance between sphingomyelins and pro-apoptotic ceramides in men who reported abnormal scores. However, metabolism of some lipid pathways tended to be divided across the BSI subscales, which may reveal important pathways that are unique to one or two of the subscales but not representative of the overlap across all three. Higher levels of acyl carnitine metabolites were observed in men with abnormal depression and phobia scores, but the opposite direction of effect estimate was observed for anxiety, suggesting that depleted levels of these metabolites may be associated with worse scores in anxiety. Carnitine is often supplemented to alleviate depression [47], but our results were not consistent with the beneficial effects of carnitine supplementation observed in previous studies. This could be due to the fact that our study relied on self-reported scores while previous findings involved comparisons of metabolites with discrete clinical diagnoses [48].

This study had strengths and weaknesses. We evaluated associations between metabolites and depression and anxiety through use of a self-report instrument (i.e., BSI scores) rather than psychiatric clinical diagnoses, which may better align with recent priorities set by the NIMH. However, some studies have found discordance between the self-evaluation of mental health conditions and clinical diagnoses [49]. This may be particularly likely to occur when self-reported symptoms are of sub-clinical intensity and, thus, are not captured by discrete clinical diagnoses. While this study aimed to evaluate mental health in these dimensions outside of strict clinical criteria, the discordance in self-evaluated scores compared to psychiatric diagnoses could also call into question the validity of the findings. BSI items only inquire about an individual’s mental health status over the previous 30 days, so it is difficult to assess whether the relationships observed were reflective of acute or chronic mental health states. Nevertheless, the dynamic nature of the metabolome reflects time-sensitive changes in physiological state, potentially reflecting disease-related biological changes occurring at the time the individuals completed the BSI assessment. Our study was underpowered to investigate any associations between metabolites and BSI dimensions with regard to race, considering that 400 of the 405 included men were White. Some studies have found differences between metabolite profiles across races and ethnicities for other diseases [50,51], which could limit the generalizability of these findings. Furthermore, the use of global metabolomics was advantageous in this exploratory study that sought to categorize changes across a multitude of possible pathways. However, due to the nature of global metabolomics as an untargeted approach, we were limited by the relatively quantified data. We were also limited to samples suitable for metabolomic profiling, which excluded samples collected prior to 2000 due to the storage conditions making these samples unsuitable for metabolomic profiling. Future studies using targeted metabolomics platforms with absolute quantification could provide improved understanding of the roles of these metabolites and pathways. Our findings are exploratory and hypothesis-generating; no metabolites retained significance after correction for multiple testing. However, despite these metabolites not surviving multiple testing corrections, the nominally significant metabolites we identified have also been identified in the previous literature. There may have been reduced statistical power to detect these associations due to the generally good health of the sample, with ≤10% scoring in the abnormal range on the subscales of interest, which could limit our ability to detect differences between individuals with normal and abnormal scores in these dimensions. Conducting a study among participants with more heterogeneous mental health phenotypes would improve the power to detect associated metabolomic alterations.

## 5. Conclusions

In this exploratory study, we discovered consistently altered metabolites across the depression, anxiety, and phobic anxiety subscales measured by the BSI. The findings provide preliminary data regarding potential biological drivers and mechanisms underlying symptoms of depression, anxiety, and phobic anxiety symptoms, as well as their overlap. Our findings suggested four metabolites that were commonly associated with abnormal scores for the three BSI subscales of depression, anxiety, and phobic anxiety and, thus, implicated them as potential biomarkers of interest for future study. This study demonstrated the utility of metabolomics in uncovering distinct and overlapping pathways observed across multiple symptom dimensions and showed that the findings are biologically translatable. Future studies targeting these pathways could provide additional mechanistic information to understand how alterations in these pathways give rise to poor mental health.

## Figures and Tables

**Table 1:** Characteristics of the 405 men from the Normative Aging Study metabolomic cohort included in analyses.

Characteristics	
Age (years), Mean [SD]	74.98 [6.61]
BMI (kg/m^2^), Mean [SD]	27.65 [4.14]
Race, N (%)	
White	400 (98.8%)
Black	3 (0.7%)
Hispanic White	2 (0.5%)
Health, N (%)	
Excellent	52 (12.8%)
Good	252 (62.2%)
Fair	71 (17.5%)
Poor/Very Poor	12 (3%)
Missing	18 (4.4%)
Income, N (%)	
Less than USD 25,000	54 (13.3%)
USD 25,000-USD 50,000	130 (32.1%)
USD 50,000-USD 75,000	83 (20.5%)
More than USD 75,000	80 (19.8%)
Missing	58 (14.3%)
Ever Married	
No	14 (3.5%)
Yes	379 (93.6%)
Missing	12 (3.0%)
Smoking Status, N (%)	
Never Smoked	61 (15.1%)
Ex-Smoker	315 (77.8%)
Current Smoker	18 (4.4%)
Missing	11 (2.7%)
Drinking Status, N (%)	
Non-Drinker	61 (15.1%)
Light Drinker	189 (46.7%)
Heavy Drinker	108 (26.7%)
Other	11 (2.7%)
Missing	36 (8.9%)
Employment Status, N (%)	
Employed	22 (5.4%)
Unemployed/Retired	355 (87.7%)
Missing	28 (6.9%)
Abnormal Depression Score, ^1^ N (%)	41 (10.1%)
Depression Score, Mean [SD]	0.26 [0.47]
Abnormal Anxiety Score, ^1^ N (%)	32 (7.9%)
Anxiety Score, Mean [SD]	0.24 [0.44]
Abnormal Phobic Anxiety Score, ^1^ N (%)	21 (5.2%)
Phobic Anxiety Score, Mean [SD]	0.12 [0.37]

^1^ Abnormal scores were categorized as scores that were one standard deviation above the sample mean, using methods applied previously as described in [24].

**Table 2:** Metabolites significantly associated (*p* < 0.05) with abnormality in all three BSI subscales of interest. The odds ratios (ORs), lower and upper 95% confidence interval (CI), and *p*-values are shown.

Metabolite	Super Pathway	Sub Pathway	Depression (N = 41 Men)				Anxiety (N = 32 Men)				Phobic Anxiety (N = 21 Men)			
			OR	Lower 95%	Upper 95%	*p*	OR	Lower 95%	Upper 95%	*p*	OR	Lower 95%	Upper 95%	*p*
Homocitrulline	Amino Acid	Urea cycle; arginine and proline metabolism	2.49	1.33	4.66	0.004	2.32	1.17	4.58	0.01	2.86	1.29	6.31	0.009
3-hydroxy-2-ethylpropionate	Amino Acid	Leucine, isoleucine, and valine metabolism	2.81	1.18	6.78	0.02	4.02	1.53	10.87	0.005	4.64	1.45	15.40	0.01
Glucuronate	Carbohydrate	Aminosugar metabolism	1.91	1.06	3.36	0.02	2.52	1.36	4.59	0.002	2.87	1.41	5.70	0.003
2-aminophenol sulfate	Xenobiotics	Chemical	1.87	1.08	3.31	0.03	2.13	1.14	4.09	0.02	2.59	1.19	5.92	0.02

**Table 3:** Selected pathways represented by plasma metabolite associations with abnormal scores on the depression, anxiety, and phobic anxiety subscales. Thirteen sub-pathways demonstrated nominally significant associations (*p* < 0.05) between at least one metabolite and abnormal scores on all three subscales of interest, despite a lack of overlap in individual metabolites. The number of metabolites in each pathway nominally associated with each subscale is shown, and individual associations can be found in Supplementary Table S1.

Super Pathway	Sub Pathway	Depression	Anxiety	Phobic Anxiety
Amino Acid	Alanine and Aspartate Metabolism	2	1	1
Amino Acid	Glutamate Metabolism	2	1	1
Amino Acid	Leucine Isoleucine and Valine Metabolism	5	1	1
Amino Acid	Methionine, Cysteine, SAM, and Taurine Metabolism	2	4	3
Amino Acid	Tyrosine Metabolism	1	2	3
Amino Acid	Urea cycle; Arginine and Proline Metabolism	3	2	2
Xenobiotics	Chemical	2	2	1
Xenobiotics	Food Component/Plant	4	3	4
Carbohydrate	Aminosugar Metabolism	1	1	1
Lipid	Fatty Acid Metabolism (Acyl Carnitine)	2	9	3
Lipid	Fatty Acid Dicarboxylate	1	3	2
Lipid	Phosphatidylethanolamine (PE)	7	1	1
Lipid	Sphingomyelins	3	2	22

**Table 4:** Mean standardized metabolite levels among men with no abnormality, individuals scoring in the abnormal range in only one of the three subscales of interest, and individuals scoring in the abnormal range on all three subscales of interest. The trends in the mean z-scored metabolite levels for each metabolite are colored from the lowest to highest z-score between the endpoints, with the no color denoting the lowest value and the darkest shade of blue denoting the highest value. Nominally significant differences are noted by bolded and italicized eta-squared and *p*-values. Eta-squared values are shown for the ANOVA of each metabolite. The 13 men with abnormality in only 2 of 3 subscales were excluded.

Metabolite	No Abnormality (N = 346)		Depression Only (N = 21)		Anxiety Only (N = 10)		Phobic Anxiety Only (N = 4)		All (N = 11)		*η^2^*	*p*-Value
	Mean	SD	Mean	SD	Mean	SD	Mean	SD	Mean	SD		
Glucuronate	−0.04	0.47	−0.11	0.26	−0.02	0.55	0.31	1.03	0.68	0.81	***0.065***	***<0.001***
Homocitrulline	−0.08	0.50	−0.02	0.46	0.02	0.40	0.00	0.93	0.58	0.71	***0.046***	***0.001***
2-aminophenol sulfate	−0.06	0.63	−0.01	0.62	0.08	0.62	−0.21	0.74	0.57	0.43	***0.030***	***0.020***
3-hydroxy-2-ethylpropionate	−0.05	0.38	−0.01	0.31	0.06	0.29	0.29	0.31	0.22	0.44	0.023	0.065

Note: Individuals are categorized as having an abnormal score on a given BSI subscale if their score is one standard deviation above the mean, as previously described in [24,26,27].

## Data Availability

The data presented in this study are available upon request. The data are not publicly available due to the inclusion of information that could compromise the privacy of the research participants.

## Supplementary Materials

The following supporting information can be downloaded at: https://www.mdpi.com/article/10.3390/metabo13070851/s1. Figure S1: Correlations between levels of metabolites associated with 3 BSI dimensions. Pearson correlation coefficients are shown for significantly correlated metabolites across the 4 total metabolite that were significantly associated with abnormality in BSI dimensions of depression, anxiety, and phobic anxiety at a nominal threshold of *p* < 0.05; Figure S2: Comparison of direction of effect and statistical significance in metabolites associations across base and maximally adjusted models for abnormality in BSI depression domain; Figure S3: Comparison of direction of effect and statistical significance in metabolites associations across base and maximally adjusted models for abnormality in BSI anxiety domain; Figure S4: Comparison of direction of effect and statistical significance in metabolites associations across base and maximally adjusted models for abnormality in BSI phobic anxiety domain. Table S1: Results for individual metabolite associations with abnormality in depression, anxiety, and phobic anxiety in base model; Table S2: Results for individual metabolite associations with abnormality in depression, anxiety, and phobic anxiety in maximally adjusted model. Refs. [28,52].
