# Supplementary material for: Metabolomics and Self-Reported Depression, Anxiety, and Phobic Symptoms in the VA Normative Aging Study
Source: Metabolites. 2023 Jul 15;13(7):851. doi: 10.3390/metabo13070851 (PMC10383599; doi:10.3390/metabo13070851)
Supplement: Supplementary file 1 [file metabolites-13-00851-s001.zip › Revised_Supplemental_06.26.23.pdf]

Supplementary Methods

Metabolomic profiling in samples from the Normative Aging Study

The Metabolon ultrahigh-performance liquid chromatography (UPLC) – tandem mass spectrometry (MS/MS) platform method has been previously described by Evans et al.[1]

Sample Preparation: Samples for metabolomic profiling were prepared using the automated MicroLab STAR® system from Hamilton Company. Recovery standards were added prior to the first step in the extraction process for QC purposes. To remove protein, dissociate small molecules bound to protein or trapped in the precipitated protein matrix, and recover chemically diverse metabolites, proteins were precipitated with methanol under vigorous shaking for 2 min (Glen Mills GenoGrinder 2000) followed by centrifugation. The resulting extract was divided into five fractions: two for analysis by two separate reverse phase (RP)/UPLC-MS/MS methods with positive ion mode electrospray ionization (ESI), one for analysis by RP/UPLC-MS/MS with negative ion mode ESI, one for analysis by HILIC/UPLC-MS/MS with negative ion mode ESI, and one sample was reserved for backup. Samples were placed briefly on a TurboVap® (Zymark) to remove organic solvent. The sample extracts were stored overnight under nitrogen prior to analysis.

QA/QC: Controls were analyzed in concert with the experimental samples: a well-characterized, pooled matrix sample generated from a representative, large number of individuals served as a technical replicate throughout the data set; extracted water samples served as process blanks; and a cocktail of QC standards that were carefully chosen not to interfere with the measurement of endogenous compounds were spiked into every analyzed sample to allow instrument performance monitoring and aid in chromatographic alignment. Instrument variability was determined by calculating the median relative standard deviation (RSD) for the standards that were added to each sample prior to injection into the mass spectrometers. Overall process variability was determined by calculating the median RSD for all endogenous metabolites (i.e., non-instrument standards) present in 100% of the pooled matrix samples. Experimental samples were randomized across the platform run with QC samples spaced evenly among the injections.

Ultrahigh Performance Liquid Chromatography – Tandem Mass Spectrometry (UPLC-MS/MS): All methods utilized a Waters ACQUITY ultra-performance liquid chromatography (UPLC) and a Thermo Scientific Q-Exactive high resolution/accurate mass spectrometer interfaced with a heated electrospray ionization (HESI-II) source and Orbitrap mass analyzer operated at 35,000 mass resolution. The sample extract was dried then reconstituted in solvents compatible to each of the four methods. Each reconstitution solvent contained a series of standards at fixed concentrations to ensure injection and chromatographic consistency. One aliquot was analyzed using acidic positive ion conditions, chromatographically optimized for more hydrophilic compounds. In this method, the extract was gradient eluted from a C18 column (Waters UPLC BEH C18-2.1x100 mm, 1.7 µm) using water and methanol, containing 0.05% perfluoropentanoic acid (PFPA) and 0.1% formic acid (FA). Another aliquot was also analyzed using acidic positive ion conditions; however, it was chromatographically optimized for more hydrophobic compounds. In this method, the extract was gradient eluted from the same aforementioned C18 column using methanol, acetonitrile, water, 0.05% PFPA and 0.01% FA and was operated at an overall higher organic content. Another aliquot was analyzed using basic negative ion optimized conditions using a separate dedicated C18 column. The basic extracts were gradient eluted

from the column using methanol and water, however with 6.5 mM Ammonium Bicarbonate at pH 8. The fourth aliquot was analyzed via negative ionization following elution from a HILIC column (Waters UPLC BEH Amide 2.1x150 mm, 1.7 µm) using a gradient consisting of water and acetonitrile with 10mM Ammonium Formate, pH 10.8. The MS analysis alternated between MS and data-dependent MSn scans using dynamic exclusion. The scan range varied slighted between methods but covered 70-1000 m/z. Raw data files were archived and extracted as described below.

Data Extraction and Compound Identification: Raw data was extracted, peak-identified and QC processed using Metabolon's hardware and software. These systems are built on a web-service platform utilizing Microsoft's .NET technologies, which run on high-performance application servers and fiber-channel storage arrays in clusters to provide active failover and load-balancing. Compounds were identified by comparison to library entries of purified standards or recurrent unknown entities. Metabolon maintains a library based on authenticated standards that contains the retention time/index (RI), mass to charge ratio (m/z), and chromatographic data (including MS/MS spectral data) on all molecules present in the library. Furthermore, biochemical identifications are based on three criteria: retention index within a narrow RI window of the proposed identification, accurate mass match to the library +/- 10 ppm, and the MS/MS forward and reverse scores between the experimental data and authentic standards. The MS/MS scores are based on a comparison of the ions present in the experimental spectrum to the ions present in the library spectrum. While there may be similarities between these molecules based on one of these factors, the use of all three data points can be utilized to distinguish and differentiate biochemicals. More than 4,500 commercially available purified standard compounds have been acquired and registered into LIMS for analysis on all platforms for determination of their analytical characteristics.

Curation: A variety of curation procedures were carried out to ensure that a high quality data set was made available for statistical analysis and data interpretation. The QC and curation processes were designed to ensure accurate and consistent identification of true chemical entities, and to remove those representing system artifacts, mis-assignments, and background noise. Metabolon data analysts use proprietary visualization and interpretation software to confirm the consistency of peak identification among the various samples. Library matches for each compound were checked for each sample and corrected if necessary.

Metabolite Quantification and Data Normalization: Peaks were quantified using area-under-the-curve. A data normalization step was performed to correct variation resulting from instrument inter-day tuning differences by registering the medians to equal one and normalizing each data point proportionately. For this study, two distinct sample sets were merged that contained disparity in the proportions of control group composition, precluding run day normalization. Accordingly, datasets were merged by setting the medians of the control groups in the two datasets to be equal. Because sampling was kept consistent across both sample sets, this mitigates concerns that this approach artificially decreases the variance of this control group by forcing the medians to be equal.

Data processing pipeline: We calculated missingness across each metabolite and each sample, according to previously utilized methods.[2] Metabolites missing ≥ 75% were excluded. Missing values were imputed as half the minimum value across all samples for each metabolite. We examined the resulting plots of principal component analysis (PCA). Interquartile range (IQR) and skewness of each metabolite were also computed. All metabolites were subsequently log-

10 transformed and pareto-scaled, and IQR and skewness were re-calculated after transformation. PCA was performed again, and distribution of PCs according to demographic variables were examined.

Supplementary Results

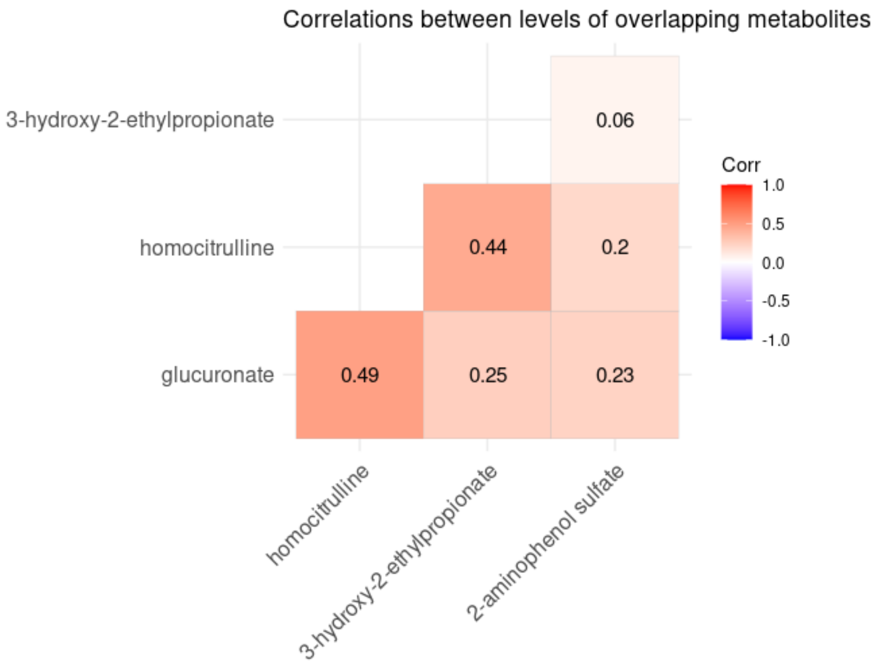

Figure S1. Correlations between levels of metabolites associated with 3 BSI dimensions. Pearson correlation coefficients are shown for significantly correlated metabolites across the 4 total metabolite that were significantly associated with abnormality in BSI dimensions of depression, anxiety, and phobic anxiety at a nominal threshold of P<0.05.

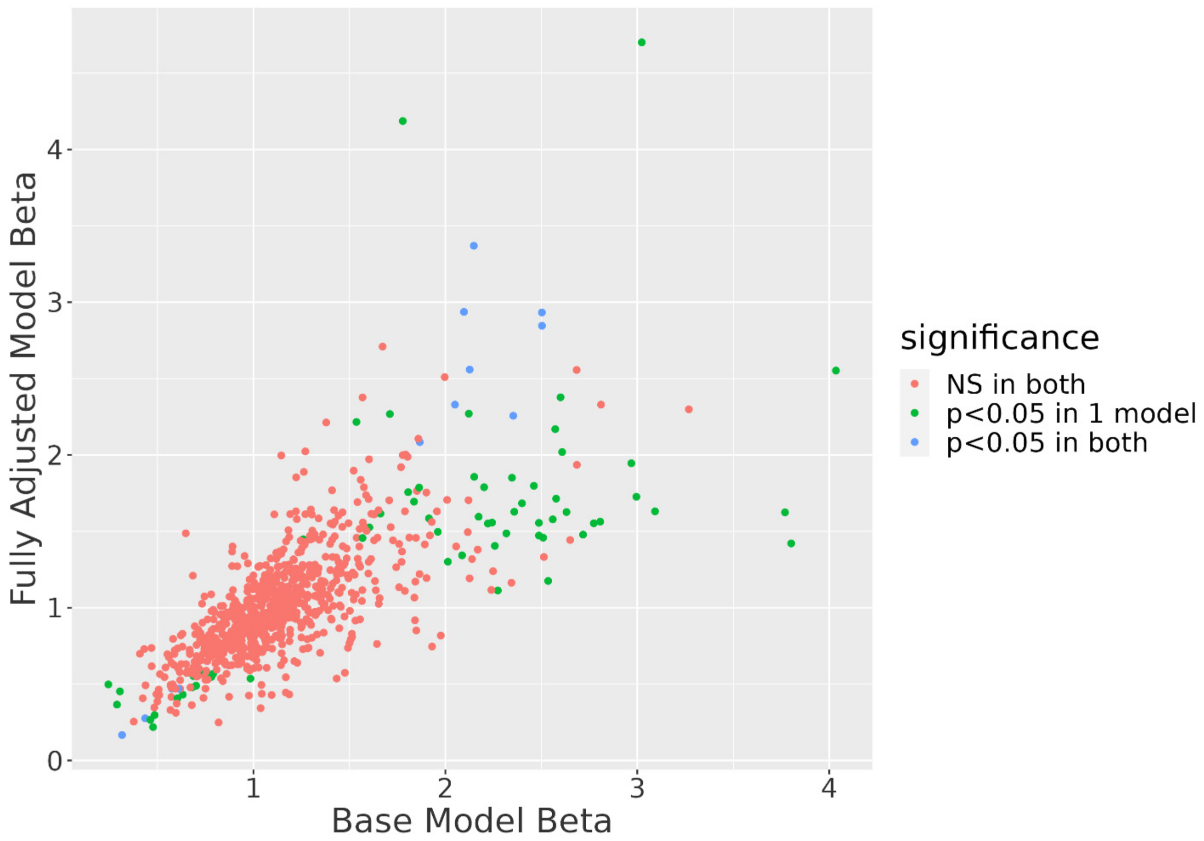

Figure S2. Comparison of direction of effect and statistical significance in metabolites associations across base and maximally adjusted models for abnormality in BSI depression domain.

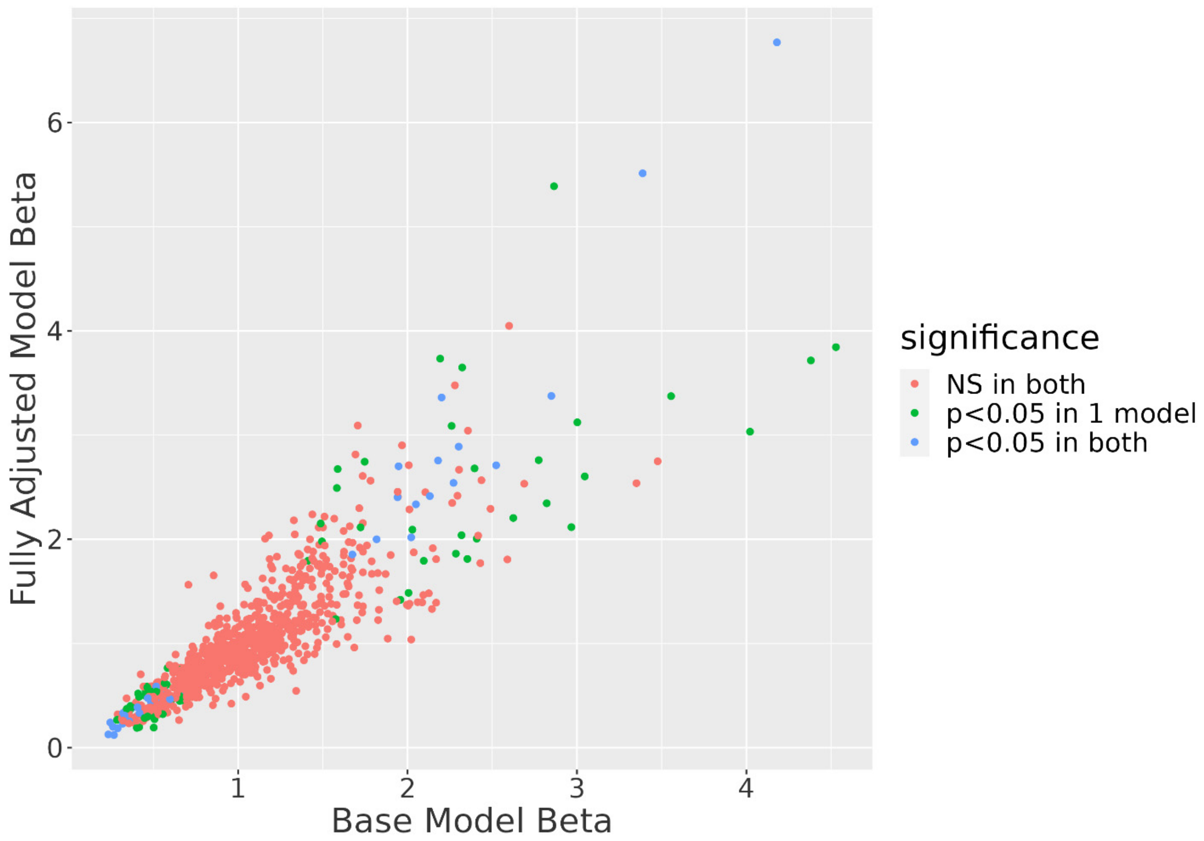

Figure S3. Comparison of direction of effect and statistical significance in metabolites associations across base and maximally adjusted models for abnormality in BSI anxiety domain.

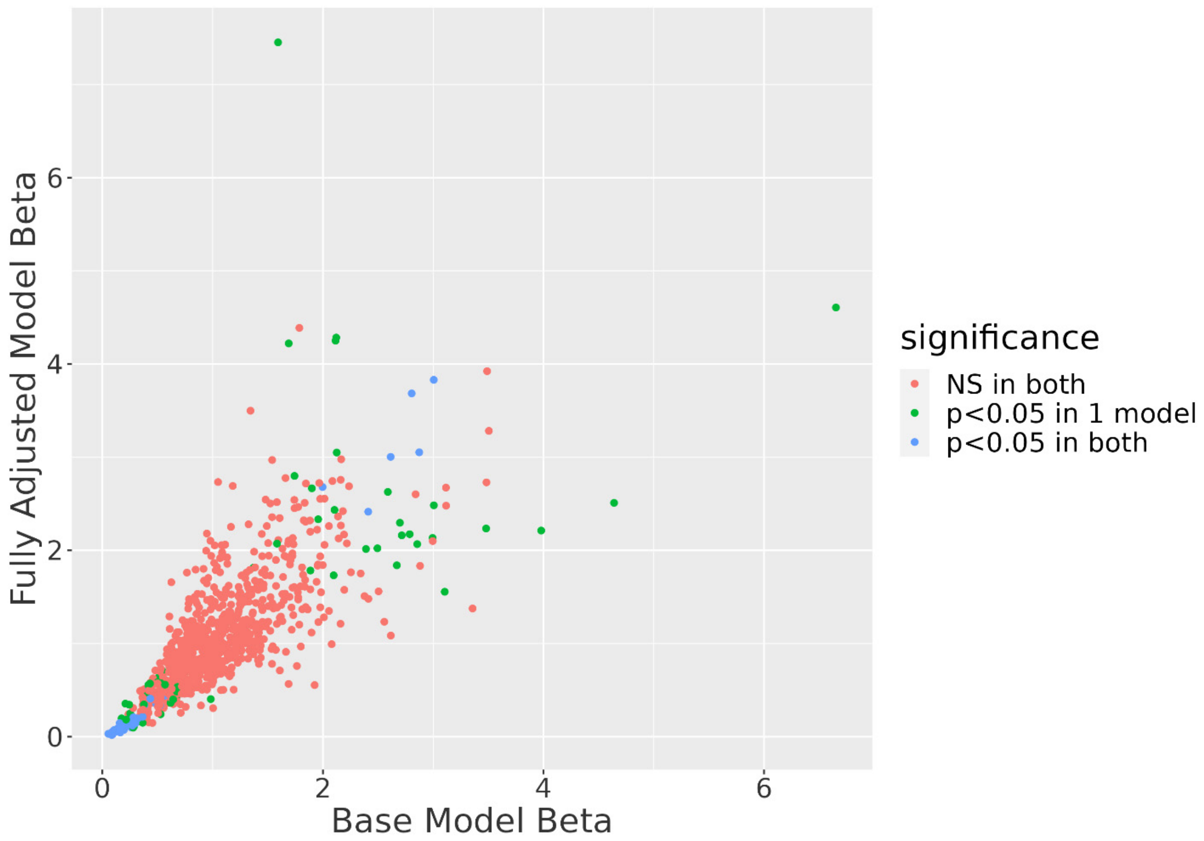

Figure S4. Comparison of direction of effect and statistical significance in metabolites associations across base and maximally adjusted models for abnormality in BSI phobic anxiety domain.

1. Evans, A.M.; DeHaven, C.D.; Barrett, T.; Mitchell, M.; Milgram, E. Integrated, nontargeted ultrahigh performance liquid chromatography/electrospray ionization tandem mass spectrometry platform for the identification and relative quantification of the small-molecule complement of biological systems. *Anal Chem* **2009**, *81*, 6656-6667, doi:10.1021/ac901536h.
2. Huang, M.; Kelly, R.S.; Chu, S.H.; Kachroo, P.; Gürdeniz, G.; Chawes, B.L.; Bisgaard, H.; Weiss, S.T.; Lasky-Su, J. Maternal Metabolome in Pregnancy and Childhood Asthma or Recurrent Wheeze in the Vitamin D Antenatal Asthma Reduction Trial. *Metabolites* **2021**, *11*, doi:10.3390/metabo11020065.
